# Evaluation of Polymer-Based Dust Palliatives in Soil and Stormwater Runoff in an Arid Environment

**DOI:** 10.1007/s00244-025-01153-6

**Published:** 2025-09-06

**Authors:** Angela P. Paul, Erin Orozco-Whitaker, Sareeha Shamsuddin, Peng Xiang, Eric Landry, Boris Poff, Solmaz Karadoust

**Affiliations:** 1 Nevada Water Science Center, US Geological Survey, Carson City, NV USA; 2 Nevada Water Science Center, US Geological Survey, Boulder City, NV USA; 3 US Bureau of Land Management, Las Vegas, NV USA; 4 PolyAnalytik, Inc., London, ON Canada

## Abstract

Dust palliatives are used to reduce fugitive dust in areas susceptible to erosion by wind and rain. In 2015, the Bureau of Land Management (BLM) temporarily approved the use of polymer-based dust palliatives during the construction and operation of a solar energy facility and, in 2019, on a mining access road in Clark County, Nevada. The areas treated with palliative are habitat to the desert tortoise. The desert tortoise consumes water opportunistically from puddles, saturated soils, or by the collection of precipitation on their carapaces. Since little is known about the toxicity of polymeric substances to the desert tortoise, the BLM is concerned with the exposure of the desert tortoise to palliative in stormwater runoff. The BLM collaborated with the US Geological Survey (USGS) to evaluate the transport of butyl acrylate vinyl acetate (BA-VA), the copolymer ingredient in the dust palliatives applied in the study, away from areas of application. BA-VA concentrations were measured in soils treated with palliative up to 90 days post-treatment, after which the copolymer became undetectable (< 0.55 mg/g). BA-VA concentrations in all stormwater samples within and outside treated areas were consistently below detection (< 0.20 mg/mL). Although stormwater and treated soils eroded from the solar facility application area were found to have BA-VA concentrations below detection (< 0.55 mg/g), it is likely that some BA-VA (parent or degradation product) was transported with suspended material. It is also likely that the amount of BA-VA transported away from areas of application was a small fraction of that applied.

**Supplementary Information:**

The online version contains supplementary material available at 10.1007/s00244-025-01153-6.

## Introduction

**Fig. 1:**
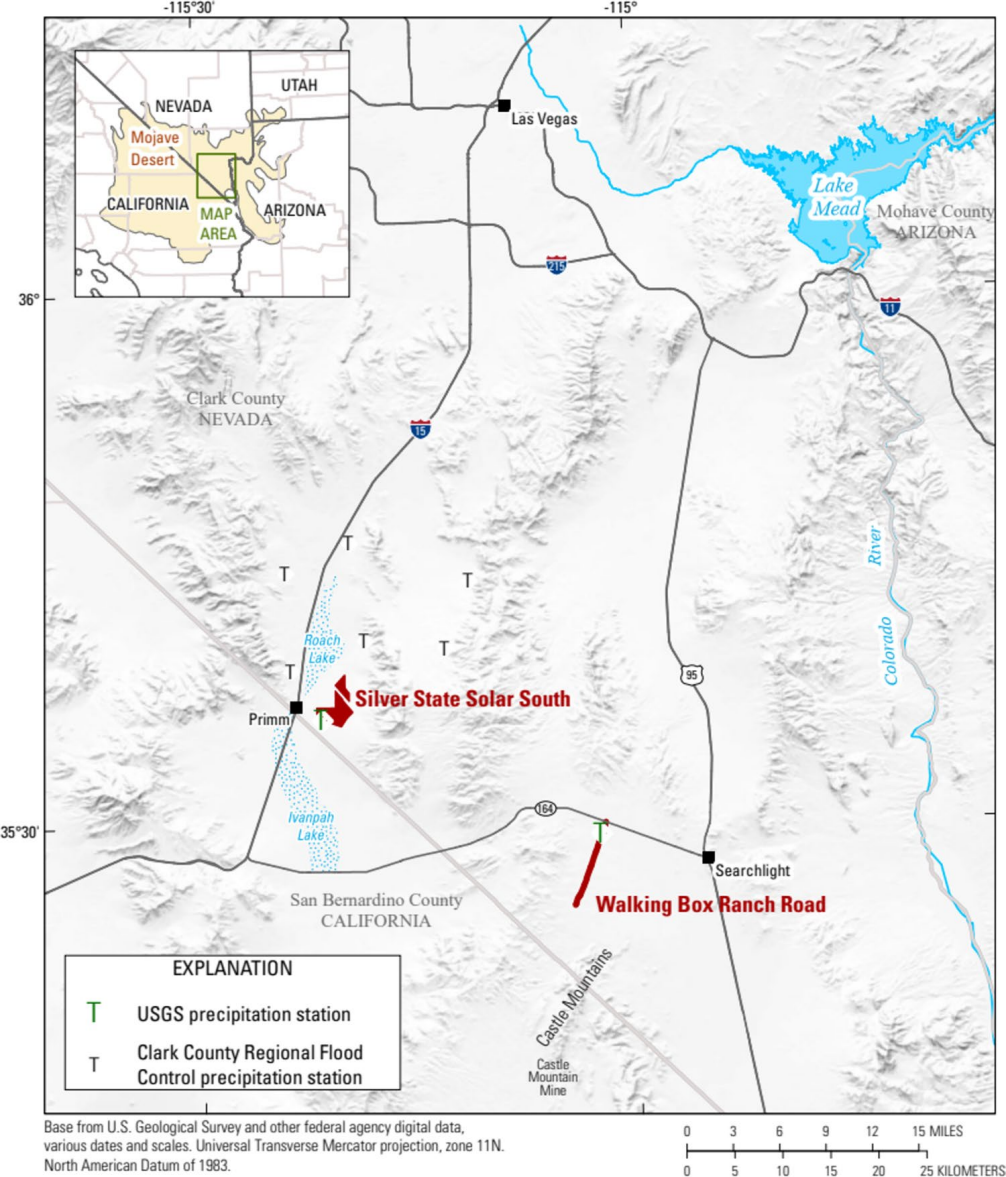
Location of the solar facility, Silver State Solar South (SSSS), the mining access road Walking Box Ranch Road (WBRR), and Clark County-maintained rain gages. USGS-maintained rain gages used for this study were located within or adjacent to site boundaries.

In 2015, the BLM approved, on a temporary basis, the use of soil palliatives (FSB 1000 and Soil Sement^®^) for the abatement of dust during the construction and operation of a solar energy facility and, in 2019, on a mining access road in Clark County, Nevada (Fig. 1); all habitat of the threatened desert tortoise, *G. agassizii* (U.S. Fish and Wildlife Service 2022; Bureau of Land Management 2013). Because of water scarcity, the desert tortoise opportunistically consumes water from puddles where rainwater has collected and by scraping through saturated soils or by the collection of precipitation on their carapaces (Esque et al. 2014).Tortoises could potentially be exposed to palliative chemicals through the ponding of stormwater in areas downgradient from areas of application during storm events causing erosion and transport of treated soils away from those areas. Manufacturers of each of the palliative formulations have reported the formulations to be composed of the copolymer BA-VA and water (Midwest Industrial Supply Inc 2001; Soil Tech 2012). The BLM collaborated with the USGS to evaluate the possible migration of BA-VA away from areas where it had been applied because of concerns the agency have regarding exposure of the desert tortoise to stormwater that may contain copolymer.

When applied, dust palliatives adhere to soils creating a crust that is resistant to physical erosion processes. Palliative transport away from areas of treatment can result from runoff, leaching, deposition of palliative laden dust, and release from tires after traversing treated areas (Bolander and Yamada 1999). The palliatives approved for temporary use by the BLM that are evaluated as part of this study are formulations FSB 1000 and Soil Sement^®^ (Table 1). The active ingredient (binding agent) in both palliative formulations is BA-VA (Midwest Industrial Supply Inc 2001; Soil Tech 2012); however, not all substances contained in dust palliative formulations are necessarily reported (U.S. Environmental Protection Agency 2004; Kunz and Little 2015; Kunz et al 2022).

**Table 1:** Primary chemical constituents in dust palliatives approved for temporary use by the Bureau of Land Management (BLM) for dust abatement during the construction and operation of a solar energy facility and on an established mining access road in southern Nevada. Both formulations contain water (CAS 7732-18-5) in various amounts.

BLM approved palliative	Characteristic chemical components in palliative	CAS number of chemical	Estimated contribution of chemical in palliative (percent weight)	Water solubility of palliative (g/L)
FSB 1000 ^a^	Butyl acrylate vinyl acetate polymer	25,067-01-0	35–50	Insoluble ^b^
Soil Sement^® ^^c^	Acrylic and vinyl acetate polymer	Not available	5–50	Dilutable ^d^

^a^Soil Tech (2012)

^b^Delgado et al. (1989); 1:1 copolymer formulation

^c^Midwest Industrial Supply Inc. (2001)

^d^Steevens et al. (2007)

The adhesive properties of synthetic polymeric substances make them attractive options for use as dust suppressants (Bolander and Yamada 1999). Generally, binders (which dust palliatives are an example) require a period of “curing” to form strong cohesive agglomerates (Engelleitner 2001). Polymeric dispersions cure through processes such as evaporation and cross-linking, the latter occurring through cationic/anionic interactions or through free radical chemistries induced by UV radiation (Umiński 2007). During cure, polymerization of lower-molecular weight polymeric material in liquid formulation transforms into higher molecular weight cross-linked coatings (Umiński 2007). Binding agents that function through chemical mechanisms create agglomeration through chemical reactions between binder components and(or) between the binder and material (Engelleitner 2001).

Singh et al. (2003) used simulated rainfall events (0.78 inches rain/hr) to evaluate the effect Soil Sement^®^ had on runoff volume and suspended sediment concentrations. Experimental plots were located in Las Vegas, Nevada (Fig. 1), with soils characterized as gravelly fine sandy loam. Singh et al. (2003) concluded that although Soil Sement^®^ increased runoff volume by decreasing soil permeability, it reduced the suspended sediment concentrations in runoff water (1,400 mg/L) relative to untreated controls (5,758 mg/L). Irwin et al (2008) found that total suspended material decreased in runoff as the synthetic polymer EnviroKleen aged over two months. Some evaluations suggest that polymeric soil-stabilizer formulations can be susceptible to leaching, harmful to vegetation, breakdown in the presence of sunlight, and redissolve upon wetting (Bolander and Yamada 1999). Consensus exists among experts that the environmental consequences of palliative treatment depend largely on the physical and chemical properties of the palliative, how it is applied, and upon the soil and climatic characteristics at the treatment site (U.S. Environmental Protection Agency 2004). Steevens et al. (2007) found acetate and acrylic polymers to be resistant to leaching from soils and are therefore unlikely to be fully dissolved in runoff water.

The scientific literature is limited with respect to available data regarding the environmental fate, transport, and toxicological risks from exposure to dust palliatives (Kunz et al 2022). Although polymeric substances are unlikely to be dissolved in water, Kunz et al (2022) determined a median lethal dose (LC_50_) for rainbow trout exposed to Soil Sement^®^ of 1,896 mg/L. Their LC_50_ was within the range of other LC_50_ values (320–8,950 mg/L) found for this palliative formulation in private consultant reports (Kunz et al 2022). Given the relatively high LC_50_ determined for Soil Sement^®^ (> 100 mg/L), Kunz et al (2022) categorized Soil Sement^®^ as “practically nontoxic.” To date, there is no information in the literature regarding the toxicity of BA-VA to the desert tortoise.

## Description of Study Areas

The results of palliative occurrence and transport at two site locations are presented. Palliatives were temporarily approved for use in both traffic and non-traffic areas within a solar development area and along a 12-mile length of a mining access road. The focus of this study is within a 1.67-acre area within the Silver State South Solar (SSSS) facility.

### Silver State South Solar Project

SSSS is a 250-megawatt photovoltaic power plant encompassing nearly 2,900 acres of public land in Ivanpah Valley in the Mojave Desert adjacent to Primm, Nevada, approximately 40 miles south of Las Vegas Valley (Fig. 1). The facility was sited within native habitat supporting densities of approximately 1.2 to 10.4 adult tortoises/km^2^ (Hromada et al. 2020). To mitigate the loss of nearly 3,275 hectares (ha) of high-quality tortoise habitat, corridors of suitable habitat were established between each block of solar panels (Hromada et al. 2020). To prevent entry onto developed areas, tortoise barriers (fencing) had been installed around the perimeter of the facility. Soil textures vary from sandy in the lower elevations (playas) of Ivanpah Valley becoming increasingly rocky at higher elevations (House et al. 2010).

**Fig. 2:**
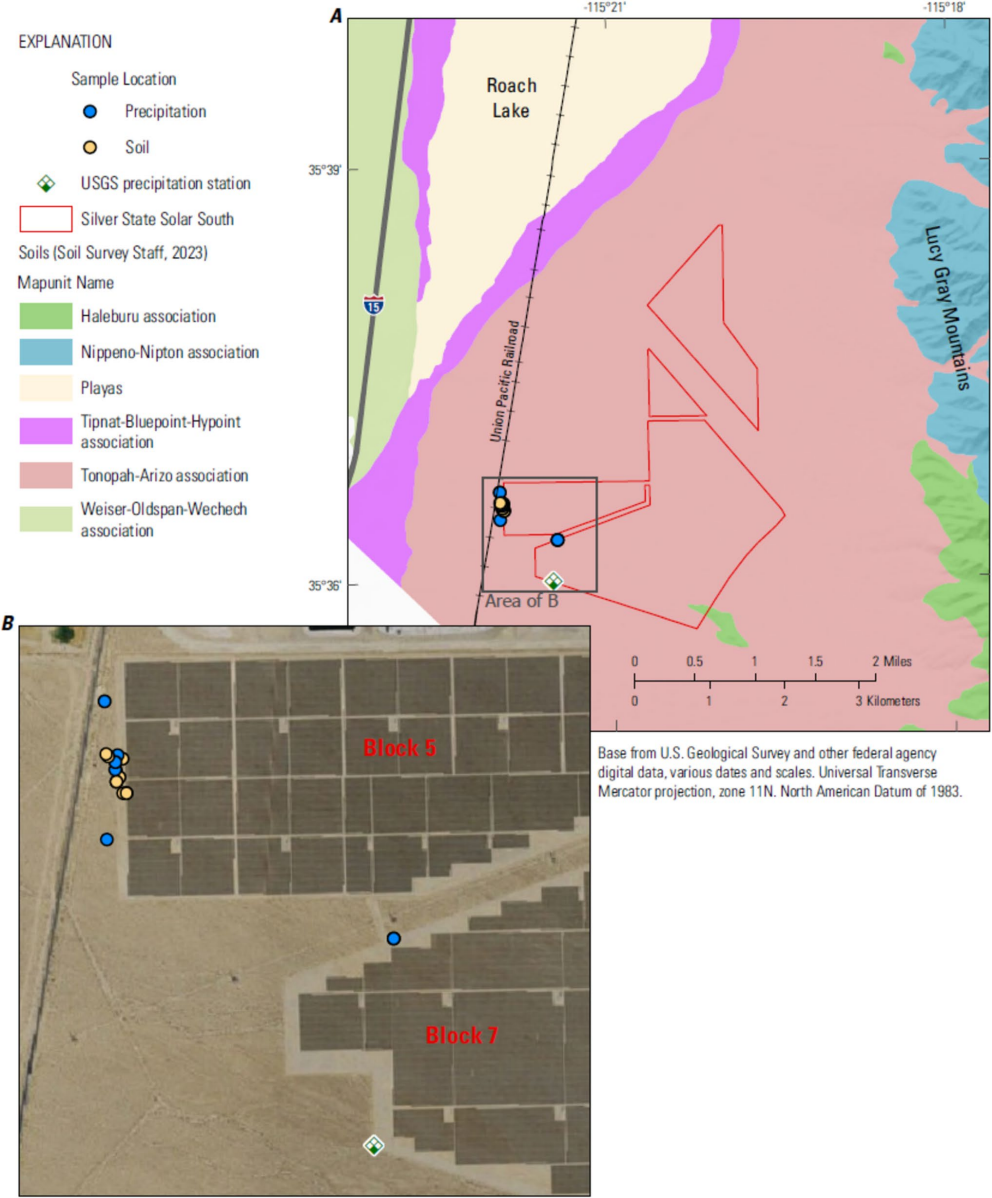
Footprint of the solar facility (and location of sample collections), soil types, and features associated with the movement of water through the Silver State Solar South (SSSS) area, southern Nevada.

Before the construction of the SSSS facility, water drained from the Lucy Gray Mountains through the alluvial fan (Fig. 2; Bureau of Land Management 2013). Retention basins were installed along the periphery of the facility to capture stormwater and designed to capture projected runoff associated with a 100-year storm event (Bureau of Land Management 2013). Suspended sediment, when transported, is expected to eventually deposit in one of the downgradient playas: Roach Lake, Nevada, or Ivanpah Lake, California (Fig. 1; Bureau of Land Management 2013).

The erodibility of undisturbed soils in the solar energy field development site was characterized by the Natural Resource Conservation Service (NRCS) as minimally erodible (Fig. 2; Table 2). Within the solar energy development, grading was required during the preparation of the land for solar panel installation. The manipulation of soils during the grading process may have changed the susceptibility of the soils to erosion.

**Table 2:** Soil characteristics and susceptibility of undisturbed soils to erosion within the footprint areas of the solar energy facility Silver State Solar South (SSSS). A dashed line indicates data unassigned to that specific soil type. All information was obtained from the US Department of Agriculture (USDA), Natural Resources Conservation Service (NRCS), Web Soil Survey database, accessed May 1, 2025, unless otherwise noted.

Site	Soil Type^a^	Soil Hydrologic Group^b^	Texture^c^	Sand/Silt/Clay (%)	Wind Erodibility Index (I)^d^	Soil Erodibility Index (K_f_)^e^
SSSS	Tonopah–Arizo association (TA)	–	Extremely gravelly sandy loam	–	8	0.05
	Tonopah (45%)	A	Sandy loam^f^	67:23:10	–	–
	Arizo (40%)	A	Loamy sand^f^	79:17:4	–	–
	Haleburu association	D	Extremely gravelly sandy loam	70:21:9	8	0.02

^a^Soil type also consists of minor components (5 to 15%) not reported in this table. The Haleburu association reported for Silver State Solar South is comprised of 85% Haleburu and 15% minor components

^b^Soil hydrologic group A is characterized by high infiltration rates and low runoff potential; soil hydrologic group D is characterized by very low infiltration rates and high runoff potential

^c^Texture provided for the topmost layer of soil as available from USDA (2025)

^d ^The wind erodibility index ranges from 1 to 8. An index of 1 is most susceptible to erosion; 8 is least susceptible to erosion

^e ^The soil erodibility factor is an index of how susceptible a soil is to erosion by water. This index ranges from 0.02 to 0.69. A higher value indicates greater susceptibility to erosion

^f ^Textures reported for specific association components were determined using the soil texture calculator (NRCS, 2025). Textures are reported for the topmost layer of soil: Tonopah–Arizo (Arizo, 0–2 inches; Tonopah, 0–1 inches); Haleburu, 0–2 inches; Colorock–Tonopah association (Colorock, 0–3 inches; Tonopah, 0–6 inches); Bard–Tonopah (Bard 0–3 inches; Tonopah, 0–6 inches)

*FSB 1000 Application*: Sometime during April 2015, newly developed areas (including Block 5) within the SSSS facility were treated with 1:10 diluted FSB 1000 palliative formulation (Table 3). Unfortunately, the analytical method required to analyze for BA-VA was still being developed during this period. On August 29, 2018, approximately 1.67 acres of exposed (free from solar panels) area within SSSS Block 5 was again regraded and treated with 1:10 diluted FSB 1000 formulation at a rate of 75 gallons/acre (Fig. 2). It was assumed that the application of palliative was uniform over the entire 1.67 area. After application with diluted FSB 1000 formulation, the treated area was divided into two sections, providing north (0.8698 acre) and south (0.8046 acre) subareas.

**Table 3:** Dates of palliative formulation application and sampling for stormwater and treated soil from each of the study areas: Silver State Solar South (SSSS) and Walking Box Ranch Road (WBRR). Abbreviations: BA-VA, butyl acrylate vinyl acetate; P, primary sample; R, replicate sample; FS, field spike; LS, laboratory spike

Site		Application date	Formulation	Area sampled within site	Samples			BA-VA Concentration
					Type	Stormwater	Soil	
SSSS	353,636,115,215,901	April 2015 ^1^	FSB 1000	Within treated area	P	–	11/08/2016	Not detected
SSSS	353,618,115,213,001	April 2015 ^1^	FSB 1000	Control, untreated area	P	02/18/2017	–	Not detected
SSSS	353,618,115,213,001	April 2015 ^1^	FSB 1000	Control, untreated area	R	02/18/2017	–	Not detected
SSSS	353,627,115,220,001	April 2015 ^1^	FSB 1000	Downgradient of treated area	P	01/09/2018	–	Not detected
SSSS	353,639,115,220,002	April 2015 ^1^	FSB 1000	Downgradient of treated area	P	07/10/2018	–	Not detected
SSSS	353,639,115,220,003	April 2015 ^1^	FSB 1000	Downgradient of treated area	R	07/10/2018	–	Not detected
SSSS	353,634,115,215,801	08/29/2018 ^2^	FSB 1000	Within treated area	P	–	08/31/2018	2.44 mg/gm
SSSS	353,634,115,215,802	08/29/2018 ^2^	FSB 1000	Within treated area	R	–	08/31/2018	3.21 mg/gm
SSSS	353,634,115,215,803	08/29/2018 ^2^	FSB 1000	Within treated area	P	–	09/28/2018	1.51 mg/gm
SSSS	353,634,115,215,804	08/29/2018 ^2^	FSB 1000	Within treated area	R	–	09/28/2018	2.54 mg/gm
SSSS	353,631,115,215,805	08/29/2018 ^2^	FSB 1000	Within treated area	P	–	11/29/2018	0.9 mg/gm
SSSS	353,631,115,215,806	08/29/2018 ^2^	FSB 1000	Within treated area	R	–	11/29/2018	1.18 mg/gm
SSSS	353,632,115,215,801	08/29/2018 ^2^	FSB 1000	Within treated area	P	11/29/2018	–	Not detected
SSSS	353,632,115,215,901	08/29/2018 ^2^	FSB 1000	Within treated area	P	02/14/2019	–	Not detected
SSSS	353,632,115,215,902	08/29/2018 ^2^	FSB 1000	Within treated area	R	02/14/2019	–	Not detected
SSSS	353,632,115,215,903	08/29/2018 ^2^	FSB 1000	Within treated area	P	–	09/06/2019	Not detected
SSSS	353,632,115,215,904	08/29/2018 ^2^	FSB 1000	Within treated area	R	–	09/06/2019	Not detected
SSSS	353,633,115,215,906	08/29/2018 ^2^	FSB 1000	Within treated area	P	11/20/2019	–	Not detected
SSSS	353,634,115,215,908	08/29/2018 ^2^	FSB 1000	Downgradient of treated area	P	12/04/2019	–	Not detected
SSSS	353,634,115,215,901	08/29/2018 ^2^	FSB 1000	Downgradient of treated area	FS	12/04/2019	–	0.87 mg/mL
SSSS	353,634,115,215,901	08/29/2018 ^2^	FSB 1000	Downgradient of treated area	R^3^	12/04/2019	–	0.76 mg/mL
SSSS	353,634,115,215,908	08/29/2018 ^2^	FSB 1000	Downgradient of treated area	LS	12/04/2019	–	0.20 mg/mL
SSSS	353,634,115,220,001	08/29/2018 ^2^	FSB 1000	Downgradient of treated area	P	–	12/04/2019	Not detected
SSSS	353,634,115,220,003	08/29/2018 ^2^	FSB 1000	Downgradient of treated area	R	–	12/04/2019	Not detected
SSSS	353,634,115,220,002	08/29/2018 ^2^	FSB 1000	Downgradient of treated area	LS	–	12/04/2019	9.43 mg/gm
WBRR	352,955,115,021,301	03/23/2020	Soil Sement^®^	Within treated area	P	04/08/2020	–	Not detected
WBRR	352,553,115,040,301	03/23/2020	Soil Sement^®^	Within treated area	P	04/08/2020	–	Not detected
WBRR	352,925,115,022,201	03/23/2020	Soil Sement^®^	Within treated area	P	04/08/2020	–	Not detected

^1 ^Entire Block 5 area treated with FSB 1000; application rate was 75 gallons per acre using 1:10 formulation dilution

^2 ^On August 29, 2018, a 1.67-acre area of Block 5 between the panel installation area and fence treated with FSB 1000

^3 ^Analytical reanalysis of the field spike

### Walking Box Ranch Road

**Fig. 3:**
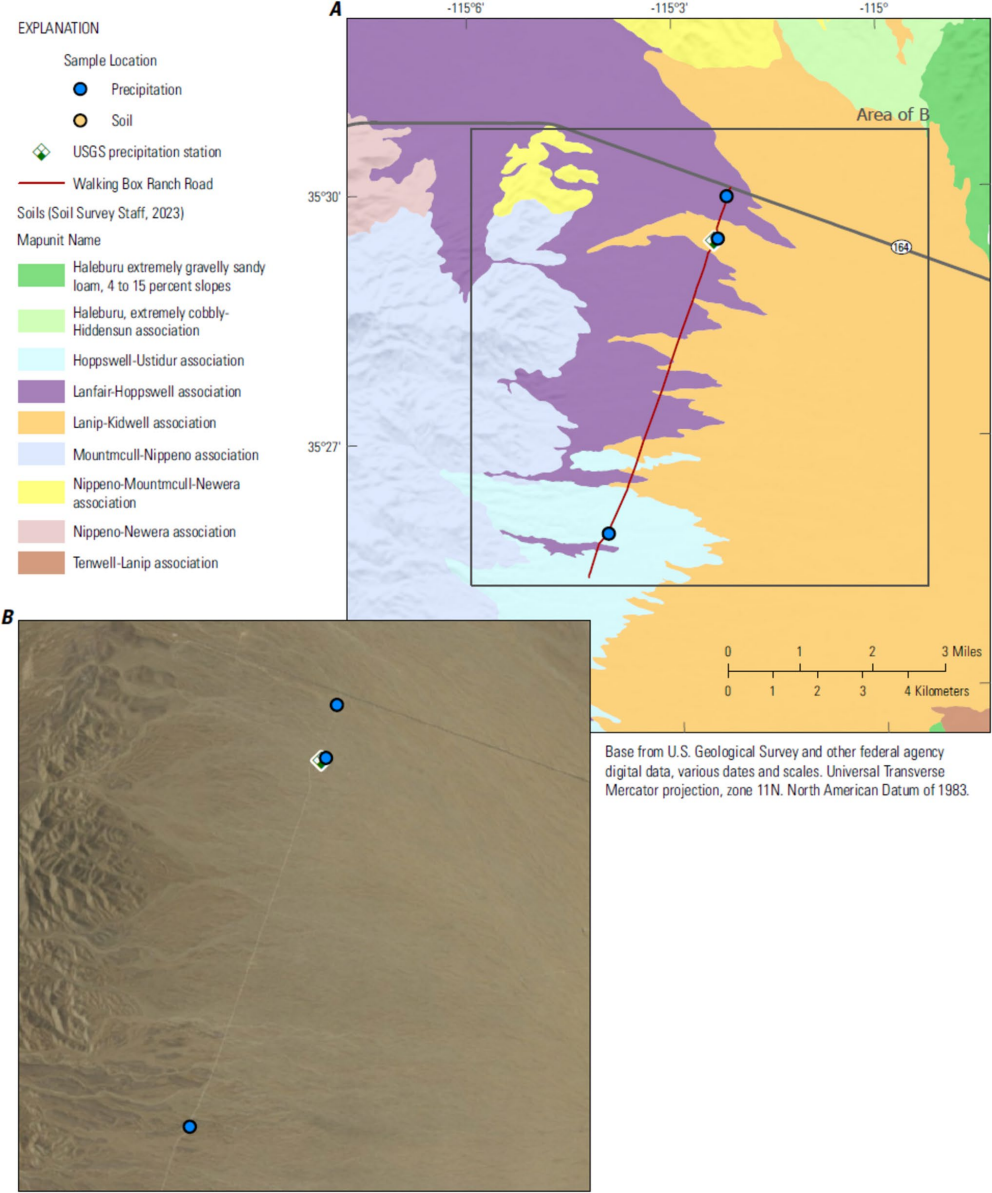
Location of site (and location of sample collections) and soil types of Walking Box Ranch Road, southern Nevada.

Castle Mountain Mine is located in the southern Castle Mountains, California, accessed by Walking Box Ranch Road (WBRR), 66 miles south of Las Vegas, 18 miles west of Searchlight, Nevada (Figs. 1, 3). WBRR already existed prior to palliative application and no grading prior to palliative application was required. To mitigate fugitive dust from the road used to access the mine, on March 23, 2020 about 12 miles of WBRR were treated with Soil Sement^®^ dust palliative.

## Methods to Determine Storm Frequency, Intensity, and Duration

**Fig. 4:**
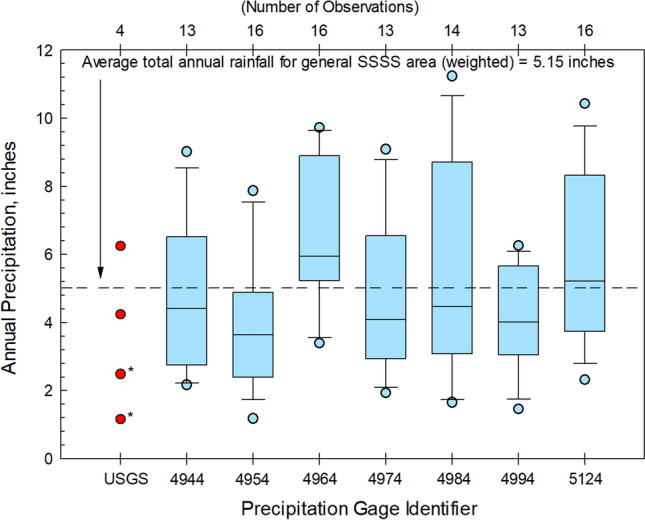
The 15-year (2005–2020) annual average rainfall in the vicinity of the solar energy facility SSSS located in southern Nevada. Data were summarized from historical rainfall data available from the Clark County Regional Flood Control District (2021). Annual USGS rain gage precipitation data collected as part of this study (USGS 353600115213201) are included for perspective; asterisk (*) indicates partial annual record. The one storm event at WBRR (USGS 352923115022501) during which stormwater samples were collected produced 0.16 inches of rain over a 1-h period (0.16 inches rain/hr).

To ensure samples collected as part of this study were collected under weather conditions typical for of the area, data obtained from USGS precipitation gages were compared to applicable regional data (Fig. 4). During winter, most precipitation in southern Nevada originates from the west; during the summer, precipitation predominately originates from the south/southeast (French 1983). The monsoon season, resulting from large shifts in wind and moisture patterns, brings large amounts of precipitation to southern Nevada and occurs from July through September (Anderson et al. 2000).

At the time of the study, there were seven rain gages operated by Clark County in the general vicinity of the SSSS and WBRR projects (Clark County Regional Flood Control District 2021; Figs. 1, 4). During the 15-year period from 2005 to 2020, available data from each Clark County rain gage were used to determine a weighted average annual rainfall. A weighted average was used to ensure annual average rainfall was not skewed toward observations obtained from any one gage. The weighted average annual rainfall for each Clark County rain gage (*WA*_*gage*_) was determined by multiplying the annual average rainfall for the gage (*µ*) by the number of observations (*n*) for that gage (Eq. 1). The weighted average annual rainfall for each study area (*WA*_*area*_) was calculated by summing all *WA*_*gage*_ values and dividing that sum by the total number of observations for all gages used in determining the weighted average, *N* (Eq. 2).

1 $$WA_{gage} = n * \mu$$

2 $$WA_{area} = \frac{{\sum WA_{gage} }}{N}$$

In addition to online storm tracking services (National Oceanic and Atmospheric Administration, various dates), tipping bucket rain gages were installed near the SSSS solar energy field development and WBRR to alert sampling personnel to storm events within the vicinity of each area (Fig. 1; Figs. 2, 3). Each rain gage (High Sierra 2400 series, 12-inch Tipping Bucket Rain Gauge) was installed and operated in accordance with USGS policy (US Geological Survey 2010). USGS rain gage data were used to quantify the amount and intensity of precipitation and to inform storm characterizations. One USGS rain gage each was installed at each site: SSSS in April 2017 and WBRR in March 2020 (Fig. 1; Figs. 2, 3). Rain gages were equipped with dual tipping buckets and an all-in-one data collection platform/geostationary environmental satellite (DCP/GOES) antenna with an internal battery mounted on a 2-inch galvanized pipe approximately 5-feet in height. The tipping buckets were calibrated to empty (tip) at 0.04 inch (1 mm) of water. The DCP was programmed to communicate hourly via GOES telemetry (real time) when tips were recorded.

## Method of Stormwater Sampling

To evaluate BA-VA transport away from areas of application, from February 2017 until April 2020, samples of unfiltered stormwater runoff were collected from two areas: SSSS and WBRR (Fig. 1). Stormwater samples were collected on-site from puddles in depressions of treated soils; downgradient areas were sampled when storm events occurred on the areal extent of the site resulting in off-site erosion and ponding (Figs. 1, 2). The timing of stormwater sampling depended upon rain events of sufficient duration and intensity to cause ponding. In addition, the solar facility was gated and sampling was dependent upon site accessibility. Generally, SSSS was accessible during normal business hours of 8 am to 5 pm, Monday through Friday.

Polytetrafluorethylene (PTFE) sampling equipment and bottles were used to prevent loss of any BA-VA present in the sample during collection, processing, and transport. Approximately one liter of stormwater was collected by either scooping water into the bottles using the PFTE bottle lid or using a 50-mL PTFE syringe, taking care not to disturb unsuspended soil within the depressions of accumulated stormwater. When enough stormwater was present within the treated area, a replicate sample was also collected using the same sampling technique as the primary (routine) sample. Sampling syringes and bottles were cleaned after each sampling event using (1) phosphate-free detergent followed by (2) tap water and (3) deionized water rinses, and (4) a final rinse with acetone and set aside to dry (E. Landry 2016, PolyAnalytik Laboratory, written communication); dry syringes and bottles were stored in clean zippered plastic bags until use. Samples were transported and stored at room temperature, in the dark, until shipped to PolyAnalytik for BA-VA analysis. Although it can be difficult to collect stormwater from natural storm events (Singh et al 2003), a total of 10 (SSSS) and 3 (WBRR) stormwater samples (including replicates) were collected as part of this study.

In addition to the samples described above, on December 4, 2019 stormwater samples were obtained outside the treated area of Block 5 of the SSSS facility (Fig. 2). The primary sample remained unspiked while the second sample was spiked in the field with 5 mL of FSB 1000 formulation. Both the unspiked and spiked samples were provided to PolyAnalytik for BA-VA analysis.

## Method of Soil Sampling

To evaluate the concentration of palliative on treated soil, soil samples were collected from the retreated 1.67-acre area within Block 5 of the SSSS facility (Fig. 2). Since it was assumed that the treatment was uniform over the entire area, samples collected from each subarea were considered replicate to the other. Primary soil samples were collected within the north subarea; replicate soil samples from the south subarea (Fig. 2). The spatial extent between the primary and replicate samples was designed to discern any variability in BA-VA soil concentration over the 1.67-acre area.

At 12 sublocations within the 1.67-acre treated area, a ruler was used to measure the depth to which the diluted FSB 1000 formulation penetrated the soil (0.5 to 2 cm); average penetration depth was 1.25 cm. Soil samples consisted of a composite of six subsamples of 10 cm by 10 cm squares (100 cm^2^) of treated soil, collected using a chisel. Each sample consisted of approximately 750 cm^3^ of soil. Initial samples of treated soil were collected each from these subareas about 48 h after treatment; the delay to allow treated soil to cure according to the manufacturer recommendation of between 24 and 72 h at 70 °F (Soil Tech 2012). After the initial cure period, treated soils were sampled 28, 90, and 371 days afterward, until BA-VA was no longer detected. Soil samples continued to be collected periodically after 371 days to evaluate if BA-VA (or any degradation products) remained below detection. During this study, a total of 11 soil samples (including replicates) were collected from within and downgradient of the 1.67-acre treated area within SSSS.

In addition to the samples described above, during the storm event on December 4, 2019 one additional soil sample was obtained just outside the fenced area downgradient of the treated area of SSSS Block 5. This sample was provided to PolyAnalytik for matrix spiking with 6.888 mL of FSB 1000 formulation.

## Method of Sample Analysis

BA-VA is not a constituent commonly analyzed in environmental matrices and, as a result, analytical method development was required. In collaboration with the BLM, PolyAnalytik, London, Ontario, Canada (PolyAnalytik Inc.) developed a gel permeation/size exclusion chromatography (GPC/SEC) analytical method to analyze samples of unfiltered stormwater and soils for the copolymer BA-VA (Shamsuddin et al. 2022). Due to the lack of a commercially available analytical standard for BA-VA, analytical precision and recovery of BA-VA from soil and water matrices were determined from extracted copolymer from each of the palliative formulations: FSB 1000 and Soil Sement^®^ (Shamsuddin et al. 2022). It is important to note that both BA-VA concentration and percent extraction recovery data from each formulation (extraction efficiency) were based on the assumption the palliative formulations contained 42.5 percent (w/w) BA-VA (the average copolymer content range reported for FSB 1000; Soil Tech 2012). Extraction efficiencies of BA-VA from FSB 1000 and Soil Sement^®^ formulations were 58 and 85 percent, respectively; GPC/SEC analytical precision 5.9 and 3.2 percent, respectively (Shamsuddin et al. 2022). Field and laboratory spike recoveries were performed on water and soil samples using FSB 1000 formulation and assuming complete matrix recovery was 58 percent (FSB 1000 formulation BA-VA extraction efficiency for method, Shamsuddin et al. 2022).

Using the formulation extraction efficiencies of Shamsuddin et al. (2022), matrix retention factors (RFs) were determined for each palliative formulation (Eq. 3a, b). The RF represents the proportion of BA-VA expected to be retained by a sample during the solvent extraction process and is a unitless parameter. The RF for each formulation was used, respectively, in calculating the minimum amount of BA-VA required in a sample to be able to quantify the concentration of the copolymer in the sample (Eqs. 4 and 5).

3a $$RF_{FSB 1000} = \left[ {1 + \left( {1 - 0.58} \right)} \right] = 1.42$$

3b $$RF_{Soil Sement\textregistered } = \left[ {1 + \left( {1 - 0.85} \right)} \right] = 1.15$$

The minimum concentration of BA-VA (C_min_) required in water and soil matrices to be measured by the analytical method developed by Shamsuddin et al. (2022) was calculated using the formulation-specific quantitation limit (Q_L_), method injection volume (Vol_*inj,*_ in mL), volume of solvent (V_*solv*_, in mL) used to extract BA-VA from the sample, RF, and the volume of water (V_*smpl*_, in mL) or mass of soil (M_*soil*_, in grams) extracted (Eqs. 4, 5*;* Table 4). The Q_L_’s for BA-VA in the FSB 1000 and Soil Sement^®^ formulations were 11.63 and 17.04 µg, respectively (Shamsuddin et al. 2022). The C_min_ of BA-VA from the application of FSB 1000 in water samples collected as part of this study ranged from 0.02 to 0.08 mg/mL; in soil matrix, C_min_ ranged from 0.52 to 2.20 mg/g. Similarly, the C_min_ of BA-VA for Soil Sement^®^ in water samples collected as part of this study was 0.20 mg/mL. The theoretical, as opposed to actual, C_min_ for Soil Sement^®^ in soil matrix ranged from 0.65 to 2.61 mg/g. Due to the compaction of soil on Walking Box Ranch Road, samples of soil treated with Soil Sement could not be collected; therefore, actual C_min_ values for soil treated with Soil Sement^®^ were not determined. C_min_ values differ between palliative formulations because of differences in formulation BA-VA extraction efficiencies, Q_L_s, and RFs.

4 $$\left( {water matrix} \right)\;C_{min} = \frac{{\left[ {\left( {\frac{{Q_{L} }}{{V_{inj} }}} \right)*\left( {V_{solv} } \right)*\left( {\frac{mg}{{1000 \mu g}}} \right)*RF} \right]}}{{V_{smpl} }}$$

5 $$\left( {soil matrix} \right)\;C_{min} = \frac{{\left[ {\left( {\frac{{Q_{L} }}{{V_{inj} }}} \right)*\left( {V_{solv} } \right)*\left( {\frac{mg}{{1000 \mu g}}} \right)*RF} \right]}}{{M_{soil} }}$$

**Table 4:** Parameters used to calculate the minimum concentration of BA-VA needed in water and soil samples to confidently identify/quantify the analyte in the sample using the analytical method developed by Shamsuddin et al. (2022)

Parameter	FSB 1000	Soil Sement^®^
Quantitation limit (Q_L_), µg	11.63	17.04
Injection volume (Vol_inj_), mL	0.100	0.100
Solvent volume (Vol_solv_), mL	50, 100 or 200	50, 100 or 200
Matrix Retention Factor (RF), unitless	1.42	1.15
Volume of water sample (Vol_smpl_), mL	200–500	200–500
Mass of soil sample (M_soil_), g	15	15

## Occurrence of Butyl Acrylate Vinyl Acetate in Stormwater Samples and Treated Soil

*Stormwater:* From 2005 to 2020, the weighted average annual rainfall in the vicinity of SSSS was 5.15 inches (Fig. 4). Excluding the two years of partial precipitation record resulting from the installation and decommissioning of the gage, the USGS rain gage at the SSSS facility indicated annual rainfall ranged from 4.12 to 6.24 inches. This amount of rainfall falls within the annual range expected for the area (Fig. 4). Tipping bucket data indicate that during each event for which stormwater samples were collected, storms yielded 0.16 to 1.4 inches (4.1 to 35.6 mm) of precipitation with storm durations from 0.25 to 7.5 hrs. Storm intensities ranged from 0.16 to 0.64 inches (4.1 to 16.3 mm) rain/hr.

In April 2015, the facility expansion at SSSS (Block 5), covering about 187 acres, was graded, prepared for solar panel installations, and first treated with FSB 1000 (Table 3). On February 18, 2017, a stormwater sample (and field replicate) was collected from an untreated corridor between Blocks 5 and 7 (Fig. 2); these control samples showed no detectable concentrations (< 0.08 mg/mL) of BA-VA.

On August 29, 2018, a 1.67-acre area along the west side of Block 5 was retreated with FSB 1000 (Table 3). From November 29, 2018 to December 4, 2019, three stormwater samples (and one replicate) were collected from within this area of SSSS (Block 5); and one stormwater sample collected downgradient of Block 5 (Fig. 2; Table 3). Storm events that resulted in the collection of these stormwater samples produced 0.16 to 1.4 inches (4.1 to 35.6 mm) of precipitation per event; storm intensities ranged from 0.16 to 0.64 inches rain/hr. On December 4, 2019, a 2-hr storm event with an average storm intensity of 0.20 inches rain/hr (0.16–0.32 inches/hr) was of sufficient duration and intensity that soil erosion was observed originating from the 1.67-acre treated area within Block 5. Although the amount of suspended material transported from Block 5 was not quantified, BA-VA would likely have been transported with eroded material. Applying select data from the simulated rain experiments conducted by Singh et al (2003) to the results of this study suggest that the amount of BA-VA that may have been transported away from treated areas (1.4 to 2.0 kg) relative to what was applied (2,004 to 2,863 kg) would have been very small (0.07%; supplemental information, “Estimated BA-VA concentration on treated soil and assessment of transport from application area”). Concentrations of BA-VA in all stormwater samples collected within and outside Block 5 were consistently below detection (< 0.08 mg/mL).

On March 23, 2020, Soil Sement^®^ was applied to about 12 miles of WBRR, used to access Castle Mountain Mine (Fig.3; Table 3). A precipitation event on April 8, 2020 (16 days post-treatment) was of sufficient intensity (0.16 inches rain/hr) and duration (one hr) to provide three stormwater samples collected from three different areas of treated surface between road mileage 0 and 10; no BA-VA was detected (< 0.20 mg/mL) in the three stormwater samples. None of the stormwater samples collected as part of this study contained measurable quantities of BA-VA. It is worthwhile to note that the minimum concentration of the copolymer in water necessary for detection (0.02 and 0.20 mg/mL) is fairly high, in the parts-per-thousand range.

*Spiked Stormwater Recovery*: To evaluate BA-VA recovery in stormwater, both a laboratory and field spike were evaluated. PolyAnalytik spiked 6.888 mL of FSB 1000 formulation into 450 mL of stormwater sample (expected recovery concentration = 3.8 mg/mL) collected outside Block 5 of the SSSS facility and allowed the spiked sample to cure under ambient light for 48 hr. When extracted and analyzed, the stormwater spike exhibited extremely low BA-VA recovery (recovered concentration 0.2 mg/mL; 5.3 percent). A stormwater sample (850 mL), replicate to the sample spiked by the laboratory, was spiked in the field with 5 mL of FSB 1000 formulation (expected recovery concentration = 1.46 mg/mL). The field spike was not exposed to ambient light before extraction and analysis and provided 55 percent BA-VA recovery (recovered concentration 0.81 mg/mL). The recovery of BA-VA from the stormwater field spike was similar to the recovery observed from extracting BA-VA from the FSB 1000 formulation (58 percent). The low recovery of the laboratory spike was attributed to the cross-linking of BA-VA polymeric chains during the 48-hr cure period (Shamsuddin et al 2022).

**Fig. 5:**
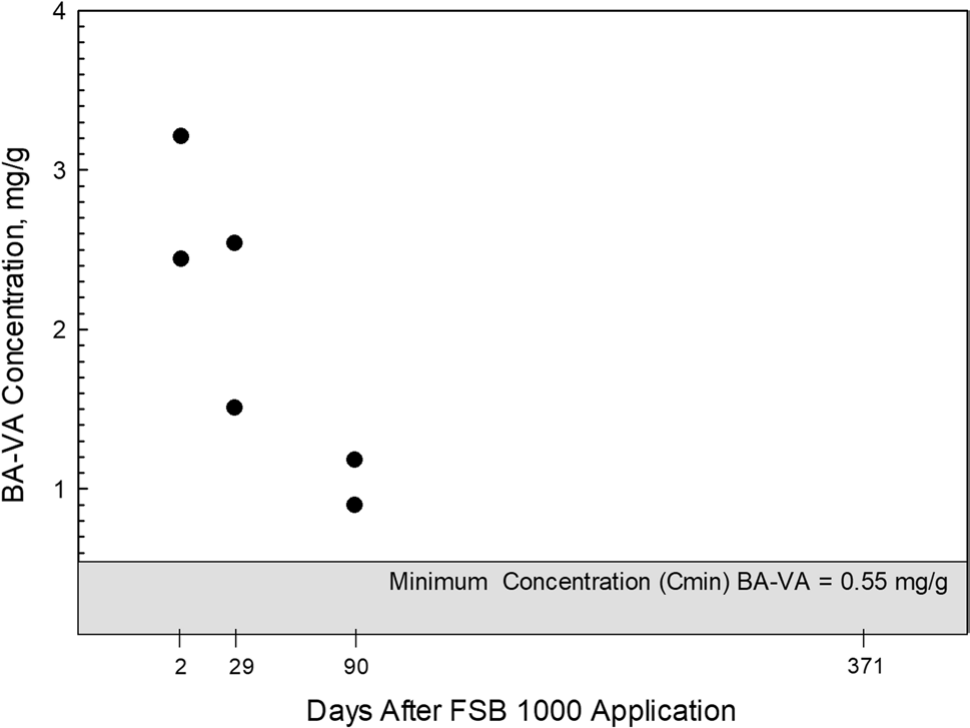
Butyl acrylate vinyl acetate (BA-VA) concentration in soil samples collected from SSSS as part of the controlled application evaluation. FSB formulation was applied, August 29, 2018

*Treated Soil:* On November 8, 2016 (about 1.5 years after the initial treatment of SSSS Block 5 with FSB 1000; Table 3), a soil sample was collected from Block 5; the sample did not contain measurable BA-VA (< 2.2 mg/g). On August 31, 2018, two days following the reapplication of diluted FSB 1000, one soil sample and field replicate were collected from the treated area (Fig. 2). BA-VA concentrations in the samples were 2.44 and 3.21 mg/g, respectively (relative percent difference = 27 percent). Over time, soil BA-VA concentrations declined eventually becoming undetectable (Fig. 5). Soil samples collected from the 1.67-acre treated area on September 6, 2019 (371 days post-application) did not contain measurable amounts of BA-VA (< 0.55 mg/g).

*Spiked Soil Recovery*: On December 4, 2019 soil samples collected downgradient of observed erosion originating from Block 5, contained no detectable BA-VA (< 0.55 mg/g). An associated soil sample collected on that same date was spiked with 6.888 mL FSB 1000 formulation by the laboratory (expected recovery concentration = 121.1 mg/g). BA-VA recovery from the spiked soil was very low (9.43 mg/g; 7.8 percent). Shamsuddin et al. (2022) suspect that after curing 48 hr under ambient light and temperature (23 °C), BA-VA spiked into water and soil may have transformed into larger molecular weight substances through cross-linking as suggested by an almost twofold to threefold increase in BA-VA weight average molecular weight (M_w_) in spiked soil and water samples, respectively, when compared to the M_w_ of uncured BA-VA extracted from the formulation (Table 3 in Shamsuddin et al. 2022; supplemental information, “Changes in butyl acrylate vinyl acetate molecular weight distributions over time after application”).

## Discussion

Stormwater sampled as part of this study remained unfiltered to mimic the water resource that would be available to the desert tortoise; no stormwater samples collected as part of this study contained measurable BA-VA (< 0.20 mg/mL). The findings of this study appear to support those of Steevens et al. (2007) in that polymeric-based palliative formulations appear to be resistant to leaching from soils. Given that acrylic palliative formulations are applied to soils specifically to adhere to and agglomerate soil particles and their low water solubility, it is not unexpected that BA-VA was not detected in stormwater samples composited from pools of water either within or proximal to treated areas. It is important to note that although concentrations of BA-VA consistently remained below detection in all stormwater samples, C_min_ values of 0.02 to 0.20 mg/mL (parts-per-thousand) are fairly high limits when compared to analytical methods developed for other polymeric substances such as per- and polyfluoroalkyl substances which can detect analytes in the parts-per-trillion range (US Environmental Protection Agency 2024).

During this study, the occurrence of BA-VA in soil treated with FSB 1000 was evaluated for a period of almost two years. The concentration of BA-VA found in treated soils decreased markedly during the first 30 days following treatment, eventually becoming undetectable (< 0.55 mg/g) after 90 days. It remains unclear whether the processes responsible for reducing BA-VA concentrations during this period were degradative; however, data suggest that after curing the BA-VA copolymer continued to chemically transform under natural field conditions at the SSSS site.

During the period of study, erosion was only noted at the SSSS solar facility and only during one event. No erosion was observed from WBRR. Previous research simulating rainfall (0.78 inches rain/hr) concluded that plots treated with acrylic palliatives exhibited reduced soil permeability to water and, as a result, increased runoff volume; however, runoff exhibited lower amounts of suspended material relative to untreated soil (Singh et al. 2003). The greatest rainfall intensity during sampling for this study (0.64 inches rain/hr) was observed at SSSS, lower than what was simulated by Singh et al. (2003). It is reasonable to assume that if any BA-VA was transported from areas of application in runoff it would likely be transported with suspended material eroded from treated areas (U.S. Environmental Protection Agency 2004). Applying select data from Singh et al (2003) to the results of this study suggest that the amount of BA-VA that may have been transported away from treated areas relative to what was applied would have been small.

Given the findings of this study, Shamsuddin et al. (2022), and other researchers evaluating BA-VA degradation (Yousif and Hadded 2013; Chelazzi et al. 2014; Gewert et al. 2015), BA-VA likely cross-links during the cure period (≤ 2 days) and continues to transform over time. Although the findings of this study largely support those of Steevens et al (2007) in that it is unlikely that BA-VA leaches into water, the desert tortoise could be exposed to BA-VA associated with the suspended material in stormwater runoff. Although it is not clear how susceptible BA-VA is to degradation and whether or not BA-VA or its degradation products are harmful to the desert tortoise, the data collected as part of this study suggest that relatively small amounts of BA-VA are likely being transported away from areas of application and concentrations were consistently below detection (< 0.20 mg/L).

## Supplementary Information

Below is the link to the electronic supplementary material.

Supplementary file1 (DOCX 585 KB)

## Data Availability

Butyl acrylate vinyl acetate concentration data for the current study are available through the data release, “Data from soil and storm water runoff samples for the evaluation of butyl acrylate vinyl acetate in an arid environment, Clark County, Nevada.” Historical precipitation data for rain gages operated and maintained by Clark County can be accessed at Clark County Nevada Regional Flood Control District. Precipitation data for USGS operated rain gages are publicly available (US Geological Survey 2021 a, b).

## Declarations

### Disclaimer

Any use of trade, firm, or product names is for descriptive purposes only and does not imply endorsement by the U.S. Government.
