# Supplementary material for: Evaluation of Polymer-Based Dust Palliatives in Soil and Stormwater Runoff in an Arid Environment
Source: Arch Environ Contam Toxicol. 2025 Sep 6;89(3):239–51. doi: 10.1007/s00244-025-01153-6 (PMC12568839; doi:10.1007/s00244-025-01153-6)
Supplement: Supplementary file 1 — Supplementary file1 (DOCX 585 KB) [file 244_2025_1153_MOESM1_ESM.docx]

# Estimated BA**-**VA Concentration on Treated Soil and Assessment of Transport from Application Area

These are the “back of the envelope” calculations describing (1) the theoretical mass of BA-VA applied to the entire 187-acre area of Block 5 within the SSSS solar facility, (2) the amount of BA-VA expected in each gram of soil collected after treatment with diluted FSB 1000 formulation and, (3) an assessment of the amount of BA-VA that may have migrated from areas of application. The calculations were made using rainfall metrics from the December 4, 2019 storm event at the SSSS site because soil erosion, thought to have originated from Block 5, was observed downgradient from Block 5 and was attributed to that storm.

The initial treatment of Block 5 occurred during Aril 2015. Diluted (1:10) FSB 1000 formulation was applied at a rate of 75 gal/acre (Boris Poff, 2016, written communication). A subsequent treatment with FSB 1000 occurred on August 29, 2018 on a 1.67-acre area near the western fence line. For the purposes of this assessment, the amount of BA-VA applied in 2018 is being considered inconsequential.

About 1,402.5 gallons of FSB 1000 (V_F_) formulation would have been required to prepare the 14,025 gallons of diluted formulation (V_d_) needed to treat the 187-acre area (equations 6 and 7). Since the FSB 1000 formulation contains 35 to 50 percent BA-VA by mass (C_P_), the 1,402.5 gallons of FSB 1000 used to prepare the diluted treatment is estimated to contain from 2,004 to 2,863 kg of BA-VA (equation 8). Assuming the palliative is uniformly applied over the entire 187 acres (756,789 m^2^), approximately 11 to 15 kg of BA-VA were applied per acre (2.6 to 3.8 g/m^2^).

$$Equation 6 V_{d}= AR*Acres$$

$$Equation 7 V_{F} = \frac{V_{d}}{DF}$$

$$Equation 8 {BAVA}_{F}= {(F}_{D} *V_{F}*C_{P})/1000$$

Where, V_d_ is the volume of diluted formulation needed to treat prescribed area in gallons

AR is the application rate in gallons per acre

V_F_ is the volume of formulation needed to prepare V_p_ in gallons

DF is the dilution factor, unitless

BAVA_F_ is the mass of BA-VA in V_F_ in kilograms

C_p_ is the decimal percent of BA-VA reported to be in the FSB 1000 formulation

F_D_ is the specific gravity of FSB 1000 (4,082 grams per gallon)

Applying a soil penetration depth of 1.25 cm over the entire 187-acre treated area of Block 5 (*see main text p. 12*), the volume of treated soil (V_soil_) is estimated to be 9,459,526,835 cm^3^. Dividing the mass of BA-VA applied to the treated area (BAVA_v_) by V_soil_ shows that each 1-cm^3^ of treated soil should contain from 0.21 to 0.30 milligrams of BA-VA (equation 9). Using a soil density of 1.6 g/cm^3^ (Prose et al. 1987), each gram of soil collected from Block 5 within SSSS should theoretically contain from 0.13 to 0.19 mg BA-VA (equation 10).

$Equation 9 {BAVA}_{v}= \frac{{BAVA}_{F}}{V_{soil}}*1,000$

$$Equation 10 {BAVA}_{soil}= \frac{{BAVA}_{v}}{D_{s}}$$

Where, BAVA_v_ is the mass of BA-VA per unit volume of treated soil in mg per cm^3^

V*_soil_* is the volume of treated soil (cm^3^)

D_s_ is the soil density in gram per cm^3^

BAVA_soil_ is the theoretical concentration of BA-VA in soil in mg per gram

Not all parameters required to assess the transport of eroded material off site were collected as part of this study. Therefore, select results from an erosion experiment by Singh et al (2003) within the Las Vegas Valley were used to surrogate those parameters. In brief, Singh et al (2003) treated experimental plots with gravelly fine sandy loam soil with a variety of dust suppressants including Soil Sement® (Table S21) which contains BA-VA (Midwest Industrial Supply, Inc., 2001).

To estimate an amount of BA-VA possibly transported during the 2-hr rain event on December 4, 2019, from Block 5 within the SSSS solar facility, a suspended sediment concentration of 1,400 mg/L was used (Singh et al, 2003). For the purposes of this evaluation, it is assumed that the application rate of dust palliative used during the Las Vegas Valley erosion experiments was the same as that of this study (as per manufacturer recommendations). It should be noted that the simulated rainfall intensity used in the erosion experiments (0.78 in/hr, Singh et al 2003) was greater than the intensities of rainfall associated with the December 4, 2019 rain event (0.16 -0.32 in/hr; average intensity 0.20 in/hr) and sample collection evaluated here.

During the December 4, 2019 storm event, a total of 0.4 inches (10.2 mm) of rain fell on the spatial extent of the 187-acre of Block 5 yielding a total volume of about 7,688,703 L (equation 11). Assuming the application of diluted FSB 1000 formulation and erosion were both uniform over the spatial extent of Block 5, using the total suspended sediment concentration (1,400 mg/L) from the Las Vegas Valley erosion study (Singh et al 2003), possibly 10,764 kg of suspended material was transported off site during that storm event (equation 12). Using the amount of BA-VA expected to be on treated soil (0.13 to 0.19 mg BA-VA/gm soil) it is possible that from 1.4 to 2.0 kg of BA-VA may have been transported downgradient from the area of application (equation 13). This amount of estimated BA-VA lost from treated areas within Block 5 accounts for about 0.07 percent of the BA-VA applied.

**Table S21** Soil characteristics and susceptibility of undisturbed soils to erosion within the footprint areas of the solar energy facility (SSSS) and the experimental plots treated with dust suppressants by Singh et al (2003). All information was obtained from the US Department of Agriculture, Natural Resources Conservation Service, Web Soil Survey database, accessed May 1, 2025, unless otherwise noted.

| **Site** | **Soil Type^a^** | **Soil Hydrologic Group^b^** | **Texture^c^** | **Sand:Silt:Clay (%)** | **Wind Erodibility Index (I)^d^** | **Soil Erodibility Index (K_f_)^e^** |
| --- | --- | --- | --- | --- | --- | --- |
| Silver State Solar South | Tonopah-Arizo association (TA) | -- | Extremely gravelly sandy loam | -- | 8 | 0.05 |
|  | Tonopah (45%) | A | Sandy loam^f^ | 67:23:10 | -- | -- |
|  | Arizo (40%) | A | Loamy sand^f^ | 79:17:4 | -- | -- |
|  | Haleburu association | D | Extremely gravelly sandy loam | 70:21:9 | 8 | 0.02 |
| Singh et al (2003), Las Vegas (NV788) | Cave-Las Vegas-Goodsprings-Destazo-Tencee | -- | Gravelly fine sandy loam | -- | -- | -- |
|  | Cave^g^ | D ^g^ | Gravelly fine sandy loam ^g^ | 65:20:15 ^g^ | 56 ^g^ | 0.20 ^g^ |

^a^ Soil type also consists of minor components (5 to 15%) not reported in this table. The Haleburu association reported for Silver State Solar South is comprised of 85% Haleburu and 15% minor components.

^b^ Soil hydrologic group A is characterized by high infiltration rates and low runoff potential; soil hydrologic group D is characterized by very low infiltration rates and high runoff potential.

^c^ Texture provided for the topmost layer of soil as available from USDA (2025).

^d^ The wind erodibility index ranges from 1 to 8. An index of 1 is most susceptible to erosion; 8 is least susceptible to erosion.

^e^ The soil erodiblity factor is an index of how susceptible a soil is to erosion by water. This index ranges from 0.02 to 0.69. A higher value indicates greater susceptibility to erosion.

^f^ Texture reported for specific association components were determined using the soil texture calculator (NRCS, 2025). Textures are reported for the topmost layer of soil: Tonopah-Arizo (Arizo, 0-2 inches; Tonopah, 0-1 inches); Haleburu, 0-2 inches; Colorock-Tonapah association (Colorock, 0-3 inches; Tonopah, 0-6 inches); Bard-Tonopah (Bard 0-3 inches; Tonopah, 0-6 inches).

^g^ U.S. Department of Agriculture (2025).

$$Equation 11 V_{R}= {1.03}^{5}*R_{i}*A$$

$Equation 12 {SS}_{e}={1.0}^{6}* SSC*V_{R}$

$Equation 13 {BAVA}_{T}= {1.0}^{-3}*{SS}_{e}*{BAVA}_{soil}$

Where, V_R_ is the volume of rainwater (L)

R_i_ is the amount of rainfall (inches)

A is the area over which the rainfall fell (acres)

SSC is the suspended sediment concentration (mg/L)

SSe is the suspended sediment from erosion (kg)

**References:**

Midwest Industrial Supply Inc (2001) Soil-Sement® Engineered Formula, Dust and Erosion Control Agent. <https://www.dustdr.com/soilsementmsds.pdf>. Accessed 13 December 2021

Prose, D.V., Metzger, S.K., Wilshire, H.G., 1987, Effects of substrate disturbance on secondary plant succession; Mojave Desert, California: Journal of Applied Ecology, vol. 24, no. 1, pp. 305-313. Also available at url, [Effects of Substrate Disturbance on Secondary Plant Succession; Mojave Desert, California (jstor.org)](https://www.jstor.org/stable/pdf/2403806.pdf)

Singh V, Piechota T, James D (2003) Hydrologic Impacts of Disturbed Lands Treated with Dust Suppressants. J Hydrol Eng 8:278-286

# Changes in Butyl Acrylate Vinyl Acetate Molecular Weight Distributions Over Time After Application

Polymeric materials are composed of repeating units (monomers) and can degrade either through bond scission, thereby lowering the average molecular weight of a material, or through crosslinking of polymeric chains, thereby increasing the average molecular weight (Yousif and Haddad 2013; Shamsuddin et al. 2022). Degradation can occur through abiotic and biologically mediated processes, generally initiated at the polymer surface (Gewert et al. 2015). Many polymeric materials absorb ultraviolet (UV) radiation rendering them susceptible to photolytic, photooxidative, and thermooxidative reactions. It appears that not only is UV radiation important for inducing adhesiveness by polymeric substances (Engelleitner 2001; Umiński 2007), but it can also influence degradation (Yousif and Haddad 2013). To be susceptible to photodegradation, the polymer must contain chromophoric groups (chemical structures with high electron density that attract UV radiation). Using nuclear magnetic resonance (^1^H-NMR) spectroscopy, Shamsuddin et al. (2022) described the general chemical structure of BA-VA. The ester groups contained within BA-VA render the copolymer susceptible to photoinduced free radical reactions and chain scissions (Gewert et al. 2015). The changes observed in the log molecular weight distribution over time in the treated soils sampled from the 1.67-acre area within SSSS (Fig. S1) appears to exemplify the processes described by Gewert et al. (2015).

Zhang et al. (2009) found that although chain scission can occur within the BA-VA polymer, vinyl acetate may stabilize polyacrylates (of which butyl acrylate is an example) from main-chain scission upon exposure to UV radiation. Duquesne et al. (2004) evaluated the thermo-oxidative degradation of vinyl acetate butyl acrylate (VA-BA) copolymers of varying vinyl acetate (VA) and butyl acrylate (BA) ratios. For the most part, VA-BA polymers were found to be more stable against thermal degradation than monomers of either VA or BA. The enhanced stability of the VA-BA copolymer was attributed to crosslinking or possibly due to changes in reaction kinetics relative to the monomeric state (Duquesne et al. 2004). Chelazzi et al. (2014) found that, in the dark, a VA-BA copolymer (65% vinyl acetate; 35% n-butyl acrylate) exhibited discoloration and morphological alteration after 168 hours exposure to 80 percent relative humidity at 80 °C. The discoloration was attributed to deacetylation, limited depolymerization, thermal rearrangement of polymer chains, and crosslinking (Chelazzi et al. 2014).

Given the findings of this study, those of Shamsuddin et al. (2022), and other researchers evaluating BA-VA degradation (Yousif and Hadded 2013; Chelazzi et al. 2014; Gewert et al. 2015), BA-VA may initially crosslink under UV-radiation (≤2 days) and subsequently undergo transformation over time. BA-VA degradation and characterization of degradation products were not specifically evaluated as part of this study, but ancillary analytical data indicate changes in the average molecular weight over time which is consistent with BA-VA transformation and degradation reactions.


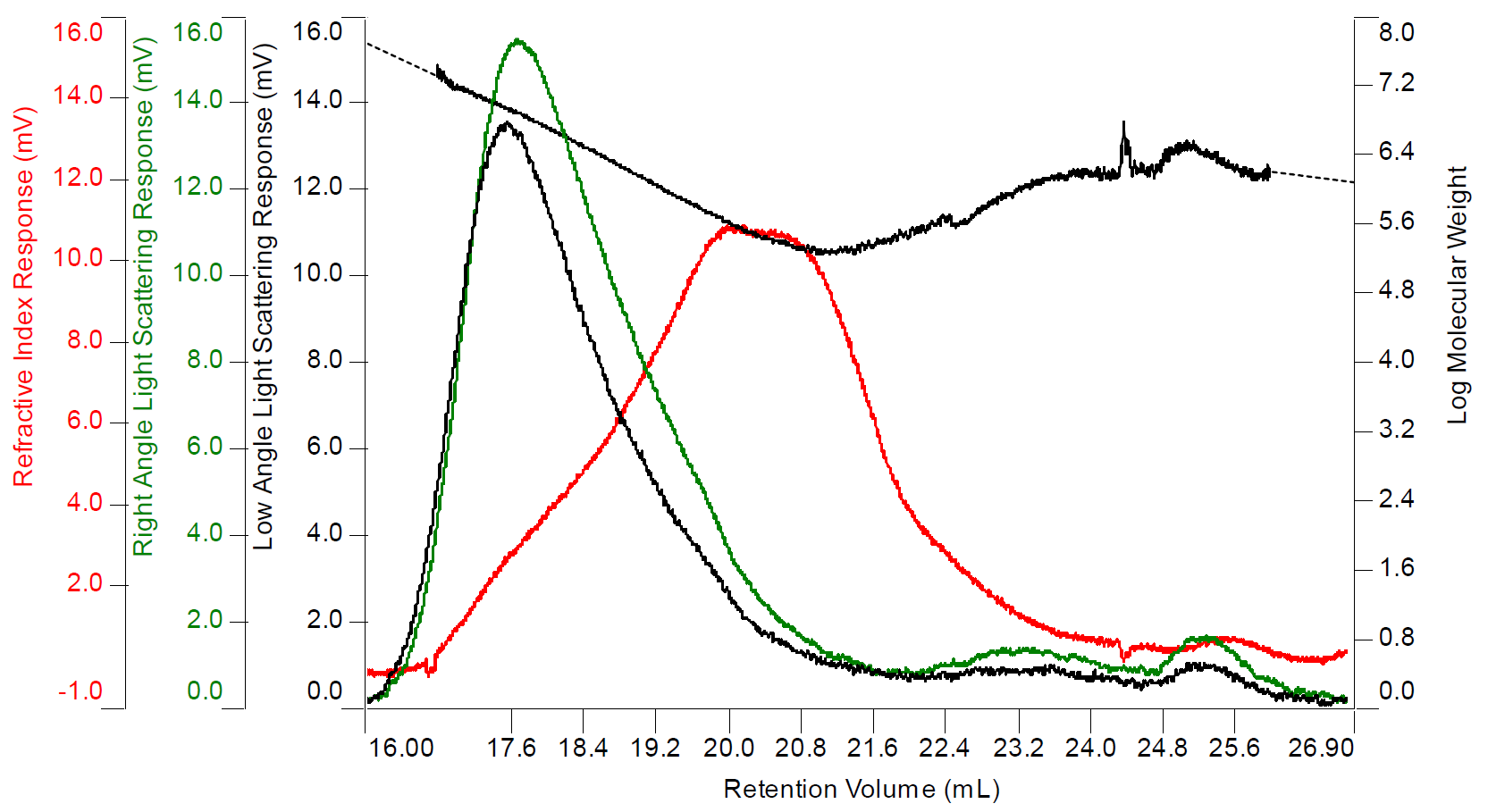


**A**


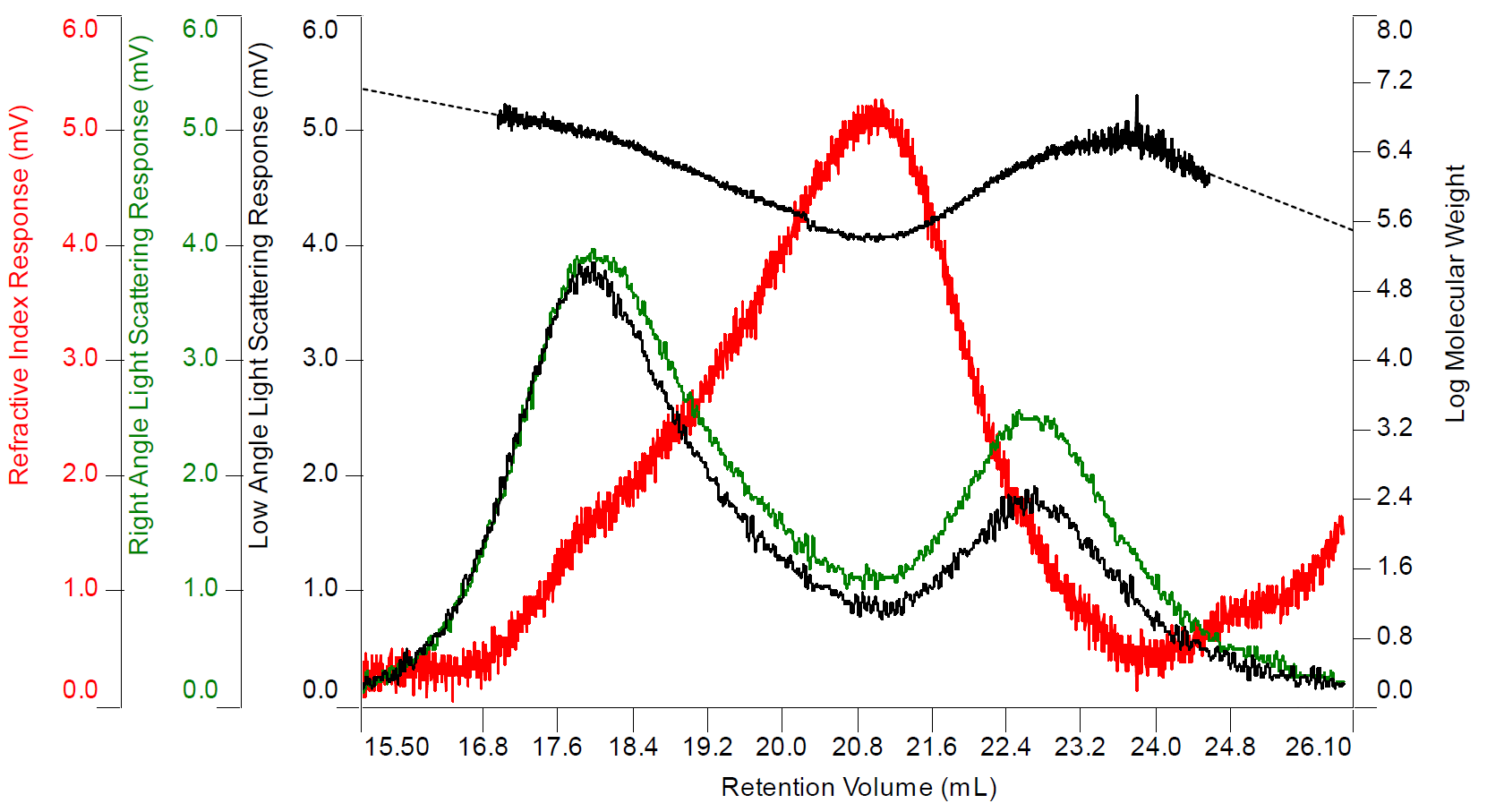


**B**


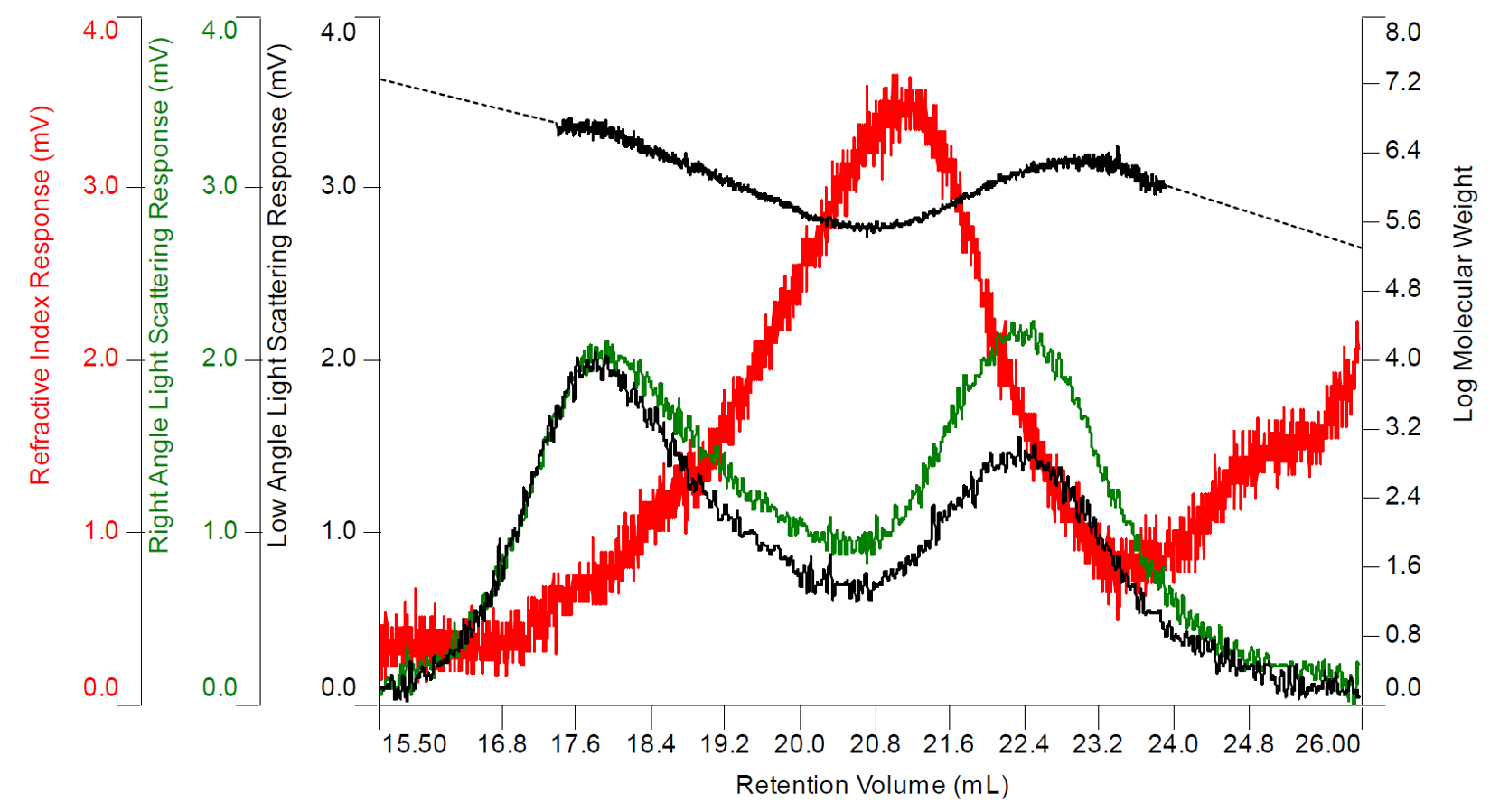


**C**

**Figure S1**. Butyl acrylate vinyl acetate (BA-VA) concentration and log molecular weight (A) 2 days, (B) 29 days, and (C) 94 days following application of FSB 1000 formulation palliative on the 1.67-acre area within Block 5 of the Silver State Solar South (SSSS) facility, August 29, 2018.

**References:**

Chelazzi D, Chevalier A, Pizzorussa G et al (2014) Characterization and degradation of poly(vinyl acetate)-based adhesives for canvas paintings. Polym Degrad Stab 107:314-320

Duquesne S, Lefebvre J, Delobel R, et al (2004) Vinyl acetate/butyl acrylate copolymers – part 1: mechanism of degradation. Polym Degrad Stab 83:19-28. <https://www.sciencedirect.com/science/article/abs/pii/S0141391003001769> Accessed 23 May 2025

Engelleitner WH (2001) Binders: How they work and how to select one. <https://www.reasearchgate.net/publication/290841282_Binders_How_the_work_and_how_to_select_one>. Accessed 12 December 2021

Gewert B, Plassmann MM, MacLeod M (2015) Pathways for degradation of plastic polymers floating in the marine environment. Environ Sci Process Impacts 17:1513-1521 <https://pubs.rsc.org/en/content/articlelanding/2015/em/c5em00207a> Accessed 23 May 2025

Shamsuddin S, Awad M, Xiang P et al (2022) Detection of the butyl acrylate-vinyl acetate (BA-VA) copolymer in soil binders using gel permeation chromatography (GPC) and nuclear magnetic resonance (NMR). Anal Lett. <https://www.tandfonline.com/doi/full/10.1080/00032719.2022.2145302>

Umiński M (2007) Environment-friendly polymeric binders: Paint Coat Ind, <http://pcimag.com/articles/87142>. Accessed 2 November 2021

Yousif E, Haddad R (2013) Photodegradation and photostabilization of polymers, especially polystyrene: review. Springerplus. <https://springerplus.springeropen.com/articles/10.1186/2193-1801-2-398>. Accessed 4 December 2022
